# The Use of Biomarkers in Early Diagnostics of Pancreatic Cancer

**DOI:** 10.1155/2018/5389820

**Published:** 2018-08-14

**Authors:** Lumir Kunovsky, Pavla Tesarikova, Zdenek Kala, Radek Kroupa, Petr Kysela, Jiri Dolina, Jan Trna

**Affiliations:** ^1^Department of Gastroenterology, University Hospital Brno Bohunice, Faculty of Medicine, Masaryk University, Czech Republic; ^2^Department of Surgery, University Hospital Brno Bohunice, Faculty of Medicine, Masaryk University, Czech Republic; ^3^Department of Internal Medicine, Hospital Boskovice, Czech Republic

## Abstract

Pancreatic ductal adenocarcinoma (PDAC) is one of the most lethal solid malignancies with increasing incidence. The poor prognosis is due to the aggressive nature of the tumor, late detection, and the resistance to chemotherapy and radiotherapy. A radical surgery procedure is the only treatment that has been shown to improve the 5-year survival rate to 20-25%. However, the majority of patients (80-85%) are diagnosed with locally advanced or metastatic disease and just 15-20% patients are diagnosed in an early stage allowing them to undergo the potentially curative surgical resection. The early detection of PDAC without the use of invasive methods is challenging and discovery of a cost-effective biomarker with high specificity and sensitivity could significantly improve the treatment and survival in these patients. In this review, we summarize current and newly examined biomarkers in early PDAC detection.

## 1. Introduction

Pancreatic ductal adenocarcinoma (PDAC) is one of the most lethal solid malignancies with increasing incidence [1] and is the fourth leading cause of cancer-related mortality in the United States and Canada [2–5].

The poor prognosis is due to the aggressive nature of the tumor, late detection, and the resistance to chemotherapy and radiotherapy [6–8]. Unfortunately, compared to other malignancies, there has been little improvement in the survival rate of patients with PDAC in recent decades [9].

The overall 5-year survival rate is approximately about 5% [2, 3, 10]. Most of the patients (80-85%) are diagnosed with locally advanced or metastatic disease. Only 15-20% are diagnosed in an early stage allowing them to undergo surgical resection [1, 11, 12]. Radical surgery has been shown to improve the 5-year survival rate to a 20-25% [12–15].

Differential diagnosis without the use of invasive methods yields difficulties distinguishing between PDAC, benign lesions, or chronic pancreatitis [16].

**Figure 1:**
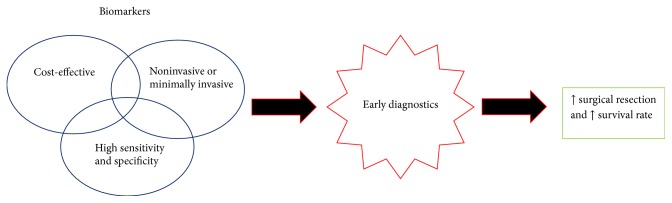
Characteristics required for biomarkers of pancreatic cancer.

As shown in Figure 1, most studies and clinical trials have sought to identify an inexpensive, noninvasive, or minimally invasive biomarker with high sensitivity and specificity for PDAC to improve early diagnosis and subsequent treatment [17]. Biomarkers for a PDAC can be classified as diagnostic, prognostic, and predictive. In this article, we will focus on a current view on diagnostic markers for early pancreatic cancer detection. The summary of possible biomarkers can be found in Table 1.

## 2. CA 19-9

The only routinely used serum marker for PDAC is carbohydrate antigen 19-9 (CA 19-9).

CA19-9 is an isolated form of Lewis antigen, which was first separated in 1979 [1, 18]. Elevation of CA 19-9 signify advanced PDAC and poor prognosis [19, 20]. However, the elevation of CA19-9 can also be caused by many other conditions, including various benign diseases (pancreatitis, cirrhosis, and acute cholangitis) [21, 22] or other malignancies (colorectal cancer, gastric, and uterine cancer) [19]. CA 19-9 is also not expressed in some individuals with a specific Lewis genotype and only 65% of patients with resectable PDAC have elevated serum levels [19, 23]. Due to all this reasons, CA 19-9 is not recommended as a screening marker for PDAC [20].

Some trials tried to improve the diagnostic value of CA 19-9 by measuring the CA 19-9 antigen on individual proteins or combining CA 19-9 with another cancer marker.

Yue et al. [24] published an article combining measurements of the standard CA 19-9 assay with detection of CA 19-9 on proteins mucin MUC5AC and MUC16; the sensitivity of cancer detection was improved relative to CA 19-9 alone in each sample set, achieving 67-80% sensitivity at 98% specificity.

Many sources confirm a better performance in the diagnosis of PDAC than CEA (carcinoembryonic antigen) measured alone [18, 21]. However, meta-analysis from 2017 showed that a combination of elevated levels CA 19-9 together with CEA (as a vital supplementary to CA19-9) can play an important role in clinical diagnosis of PDAC [25].

Lee et al. (2018) found that combined detection with CA 19-9 and cell migration-inducing hyaluronan binding protein (CEMIP) levels may have the potential to become a new laboratory indicator for the clinical diagnosis of PDAC. CEMIP exists as a newly identified protein involved in hyaluronan degradation and its increased expression has been reported in various cancers. Results suggest that CEMIP proteins were highly expressed in patients with PDAC compared to healthy individuals. Combining the use of CA 19-9 and CEMIP significantly increased the sensitivity and specificity in discriminating not only patients with all stage pancreatic cancer, but also patients with stage I/II pancreatic cancer from healthy individuals [26].

In 2015 Ritchie et al. [27] presented a new biomarker, serum fatty acid metabolite PC-594, that more clearly identified PDAC than did CA 19-9.

In summary, CA19-9 is the only routinely used serum marker of PDAC in clinical practice today. However, its elevation is usually a sign of an advanced disease and it can also be related to a variety of benign and malignant diseases other than PDAC. Thus, future studies should be directed at exploring if CA19-9 in combination with other markers can yield improved sensitivity and specificity.

## 3. Proteomics

As mentioned above, proteins such as CEMIP can be used for pancreatic cancer detection. Other proteins have been investigated as well. Sogawa et al. [28] demonstrated that the serum C4b-binding protein *α*-chain (C4BPA) level functions as a potential serum biomarker which distinguishes PDAC from chronic pancreatitis and major gastroenterological cancers, including biliary tract cancer. Yoneyama et al. [29] reported that insulin-like growth factor-binding protein (IGFBP) 2 and IGFBP3 have the ability to discriminate PDAC patients at an early stage from healthy controls. Furthermore, diagnosis of PDAC using the combination of CA19-9, IGFBP2, and IGFBP3 is significantly more effective than CA19-9 alone. This suggests that IGFBP2 and IGFBP3 may serve as compensatory biomarkers for CA19-9.

In summary, proteomics represents a promising area of research with several potentially useful proteins that seem to be able to detect PDAC even at an early stage. Future research is likely to focus on testing the role of various proteins in combination with CA19-9.

## 4. M2-Pyruvate Kinase (M2-PK)

In 2008 Novotny et al. [30] investigated M2-pyruvate kinase (M2-PK) as a potential tumor marker for distinguishing pancreatic cancer, chronic pancreatitis (ChP), and healthy controls. In group of 132 patients, a higher serum level of M2-PK was found in patients with advanced PDAC. The resulting levels were significantly higher than in patients with early PDAC, ChP, and healthy controls. Unfortunately, the differences between early PDAC and ChP were not found, which makes this marker not suitable for an early PDAC detection.

Joergensen et al. [31] sought to compare the diagnostic utility of M2-PK versus CA 19-9. The sensitivity and specificity of M2-PK were lower compared with CA 19-9 in overall PDAC detection. However, the levels of M2-PK were not affected by cholestasis or Lewis phenotype as is a limitation in the case of CA 19-9.

An additional benefit of M2-PK as a marker includes the finding that M2-PK has been associated with poorer prognosis and survival rate in those diagnosed with PDAC or periampullary malignancy [32, 33].

In summary, M2-PK has been tested as a potential marker for PDAC; unfortunately, findings reflect lower rates of sensitivity and specificity when compared with CA 19-9.

## 5. Other Metabolomic Biomarkers

Several investigators have identified some metabolomics as promising diagnostic markers for the early detection of PDAC, including palmitic acid, glucitol, xylitol, inositol, and histidine [34, 35].

The recent and largest study conducted to identify a tumor biomarker signature distinguishing PDAC from ChP using a metabolomics approach was published by Mayerle et al. [36] in 2018. They investigated 914 subjects (patients with PDAC, ChP, liver cirrhosis, and healthy cohorts) and identified a metabolic biomarker signature comprising 9 metabolites (histidine, proline, sphingomyelin d18:2, sphingomyelin d17:1, phosphatidylcholine, isocitrate, sphingagine-1-phosphate, pyruvate, and ceramide), which was used in conjunction with CA19-9 to detect PDAC with a much higher diagnostic accuracy than CA19-9 alone.

The list of metabolomics used for detection of early PDAC can be seen in Table 1.

In summary, metabolomic studies appear to represent a promising approach for the detection of PDAC at an early stage, especially when tested as a panel of several markers and in combination with CA19-9.

## 6. IgG4

High levels of immunoglobulin G4 (IgG4) in blood serum are associated with a group of autoimmune diseases called IgG4-related diseases [37]. The most common clinical manifestation is autoimmune pancreatitis (AIP) [38].

In some patients with PDAC, IgG4 elevation has been reported and it can constitute a differential diagnostic problem between AIP and PDAC. In 2012, Dite et al. [39] found in his group of 81 patients with histologically verified PDAC an elevated serum level of IgG4 (exceeding 135 mg/dl) in 8 patients. Due to this result IgG4 elevation is not suitable as a sole marker to differentiate between PDAC and AIP. However, more than twice elevated serum IgG4 level is considered to be an important diagnostic indicator, as it is present in less than 1% of PDAC patients. Some investigators explored a possible correlation between serum elevated IgG4 and pancreatic cancer. Ngwa et al. [40] reported that approximately 10% of PDAC patients have an elevated IgG4 serum level. Mild elevations in serum IgG4 are unlikely to distinguish AIP from PDAC. Serum IgG4 elevation appears to have no prognostic significance in PDAC and serum IgG4 elevation more than 2 times the upper limit appears to be most commonly associated with AIP.

In contrast to the findings reported above, a 2018 meta-analysis [41] found that serum IgG4 has high specificity and relatively low sensitivity in the differential diagnosis between AIP and pancreatic cancer, and therefore it considers serum IgG4 as useful in distinguishing AIP from PDAC.

In 2016 Liu et al. [42] published a study looking at IgG4-positive plasma cell infiltration in patients with pancreatic cancer and its correlation with the clinicopathologic traits and overall survival of pancreatic cancer. Findings suggest that high-level infiltration of IgG4-positive plasma cells may be an independent predictor for poor overall survival in PDAC patients after curative resection. The study did not investigate a possible IgG4-positive plasma cells infiltration in early diagnostics of PDAC, so this remains unclear and a potential area of further inquiry.

In summary, elevated levels of IgG4 are a typical finding in AIP; however, mild elevation can also be present in PDAC. Levels of IgG4 more than 2 times the upper limit are rare in PDAC and in conjunction with negative CA19-9 are highly suggestive of AIP.

## 7. Cytokines

In 2016, Yako el al. [2] conducted a systemic review of sixty-five studies analyzing 41 different cytokines in connection with PDAC. Six cytokines (interleukin-1*β*, interleukin-6, interleukin-8, interleukin-10, vascular endothelial growth factor, and transforming growth factor) were consistently reported to be increased in PDAC by more than four studies. However, the review did not demonstrate sufficient evidence to support individual cytokines as a diagnostic biomarker for PDAC. However, the use of panel of cytokines may be a tool for distinguishing PDAC from other pancreatic benign diseases or healthy controls.

Increased serum levels of macrophage inhibitory cytokine-1 (MIC-1), a distant member of transforming growth beta factor, in PDAC patients compared to those with benign pancreatic diseases and healthy controls were reported in some studies [43, 44]. Additionally, an elevated MIC-1 level has been shown to be related to tumor progression [2, 45].

A 2017 meta-analysis comparing 14 studies with a total of 2826 subjects has demonstrated that serum MIC-1 yields a diagnostic accuracy comparable to CA19-9 for PDAC [46].

Some studies suggest that the lack of diagnostic specificity of MIC-1 may be enhanced using a combination of MIC-1 and CA 19-9 [11, 47].

In summary, testing of cytokine levels may be beneficial in PDAC detection; however, testing of levels of individual cytokines (e.g., MIC-1) was only comparable to CA19-9. Thus, future research may focus on the use of a combined panel of individual cytokines.

## 8. Noncoding RNAs

Noncoding RNAs (ncRNAs) are divided into two groups according to their lengths, small ncRNAs (sncRNAs) (up to 200 bases), and long ncRNAs (lncRNAs) (over 200 bases) [48, 49].

Micro-RNAs (miRNAs) belong to a noncoding RNA, which do not code for proteins. They are a group of small ncRNAs (approximately 18-25 bases long) that regulate gene expression at the posttranscriptional level, through transcript degradation or translational repression. In recent years, the role of miRNA has increasingly gained attention as a potential marker for many types of cancer, including as a potential biomarker for the early detection of PDAC [11, 48]. For this reason, the miRNA has been isolated and studied from pancreatic tumor tissue, blood samples (serum, plasma), pancreatic juice, stool, urine, and even saliva.

Several studies have reported that miRNAs or panels of miRNAs identified in the plasma or serum of PDAC patients show potential diagnostic value, some of them beyond that of CA19-9 [9, 50–52].

Liu et al. [50] showed miR-1290 as biomarker able to distinguish early PDAC from healthy subjects with better diagnostic performance than CA19-9.

The largest case-control study on miRNA in patients with pancreatic diseases was conducted by Schultz et al. [51], including 409 individuals with PDAC, 25 patients with ChP, and 312 healthy controls. The authors found 9 miRNAs with diagnostic value (after testing more than 700 miRNAs), but this result was not superior to CA19-9.

In a multicenter study, Xu et al. [52] showed that miR-486-5p exhibits diagnostic value in discriminating patients with PDAC from normal subjects or patients with ChP (with a the diagnostic value comparable to CA19-9). Similar results were obtained by Le Large et al. [16], where the diagnostic potential of miR-486-5p for distinguishing PDAC from healthy controls was comparable to CA19-9.

In 2015, Vychytilova-Faltejskova et al. [53] published results showing that expression levels of miR-21, miR-34a, and miR-198 were significantly higher, whereas levels of miR-217 were significantly lower in PDAC, in comparison to healthy controls and patients with ChP.

Several studies found that miR-216 and miR-217 are downregulated in PDAC while miR-143, miR145, miR-146, miR-148, miR-150, miR155, miR-196a, miR-196b, miR-210, miR-222, miR-223, and miR-31 are upregulated in PDAC [17, 54, 55].

Hernandez et al. [6] in 2016 wrote a review article about the current knowledge on miRNA in PDAC and its precursor lesions, concluding that miR-21, miR-155, miR-196, and miR-210 are dysregulated in serum, tumor tissue, cyst fluid, and also stool of PDAC patients. In PanIN and intraductal papillary mucinous neoplasm lesions the miR-21, miR-155, and miR-196 are dysregulated as well and suggest their use as early biomarkers.

In summary, miRNAs appear promising as candidates for biomarkers of early PDAC [49], though additional studies are required for further validation of these findings. Additionally, methodology should be standardized if these approaches are ever to be used in the clinical practice [17, 48].

The use of miRNAs detected from stool, urine, pancreatic juice, and saliva will be discussed in chapter about body fluids below.

LncRNAs are restricted to specific cell types and play a crucial role during tumorigenesis by modulating key pathways at the transcriptional, posttranscriptional, and epigenetic levels [9, 56, 57]. The diagnostic value of circulating lncRNA has been demonstrated in various malignancies, such as prostate cancer, hepatocellular carcinoma, colorectal cancer, and non-small cell lung cancer [9, 58–61].

However, the association between lncRNAs and PDAC has not been well investigated, although some studies suggest they may be promising diagnostic markers. Recent studies have reported dysregulation of lncRNAs in patients with PDAC, such as H19, HOTAIR, HOTTIP, and MALAT-1 [62–65].

In 2016, Wang et al. [66] suggested that increased levels of HOTTIP were found in PDAC tissue and described this as a potential marker. In 2016, Xie at al. [67] showed that salivary HOTAIR and PVT1 distinguished PDAC patients from healthy controls and patients with benign pancreatic tumor with sensitivities and specificities ranging from 60 to 97%.

Although lncRNAs seem to possess a potential diagnostic value for early PDAC, the use of lncRNAs as a noninvasive examination modality in PDAC remains relatively uncommon. Further study on the use of lncRNAs as a potential markers is warranted.

## 9. Liquid Biopsy

The potential for the use of liquid biopsy in several malignancies (including PDAC) has been investigated, in terms of the possible role of circulating tumor cells (CTCs), circulating tumor DNA (ctDNA), and exosomes.

### 9.1. Circulating Tumor Cells (CTCs)

In 1869, Ashworth first reported the existence of circulating tumor cells (CTCs) [12, 68, 69]. CTCs are cells derived from a primary tumor that have entered the vasculature and circulate within the blood stream looking to seed in distant organs [8, 70]. CTCs appear in extremely low frequencies, approximately 1 CTC per billion blood cells in patients with a malignancy [13], and the identification and isolation in pancreatic cancer have proven difficult thus far [17]. However, several studies have shown that CTCs can enter the bloodstream in the early stages even in the case of PDAC [68, 71, 72].

In a recent trial, Kulemann et al. [73] (using a filtration-based method and KRAS [Kirsten rat sarcoma] mutational analysis) reported that CTCs can be found in most patients with PDAC of any stage (localized, locally advanced, or metastatic). They detected CTCs in 73% of patients with PDAC regardless of tumor stage. CTCs were identified in 3 of 4 patients (detection rate 75%) with early PDAC and were not detected in blood from 9 health donors.

Gao et al. [74] reported sensitivity of 88% and a specificity of 90% in patients of various stages of PDAC using subtraction enrichment and immunostaining-fluorescence in situ hybridization.

In 2016, Ankeny et al. reached a sensitivity of 75% and a specificity of 96,5% in detecting PDAC (also counted in all PDAC stages) using a different technique (microfluidic NanoVelcro CTC chip) [75].

Although CTCs seem promising in the early detection of PDAC [9], more data on the sensitivity and specificity of CTCs is needed [12, 13, 17]. A limitation of the use of CTCs as a liquid biopsy is their relatively low sensitivity, rarity and heterogeneity of CTCs, and a lack of clarity on the most effective method of detection [13, 76]. Therefore, a standardized detection method and large-scale validation are required before clinical application [9].

### 9.2. Circulating Tumor DNA (ctDNA)

Circulating free DNA, also called cell-free DNA (cfDNA), was first reported and verified by two French biochemists (Mandel and Metais) in 1948 [77]. The cfDNA consists of small double-stranded DNA fragments found in blood. In healthy people, most cfDNA is derived from bone marrow and other organs such as the liver [12]. Tumor cells also release fragments of DNA called circulating tumor DNA (ctDNA), which was firstly described in 1989 [78, 79].

The ctDNA represents a variable fraction of cfDNA, accounting for 0.01% to more than 50% of the cfDNA [80]. Due to the presence of cancer-related mutations, ctDNA can be effectively distinguished from normal cfDNA [12, 68].

In pancreatic intraepithelial neoplasms (PanINs) and PDAC, the predominant genetic characteristic is the high rate of KRAS mutations, which is directly correlated with PanINs grade [9, 12]. Due to this high frequency of KRAS mutations in PanINs and PDAC, ctDNA could be potentially used as a biomarker in detecting early PDAC. However, there is some concern regarding specificity, as KRAS mutations are not exclusive to PDAC, but are also present in various types of malignancies and even in ChP [9, 81].

Several research groups have reported that ctDNA could be detected in about 50% of early stage PDAC by digital PCR approaches [82–84].

Bettegowda et al. [84] detected ctDNA in 640 plasma samples of patients with different types and stages of cancers, including 155 PDAC patients, and showed that the detection rate of ctDNA was 48% in patients with localized PDAC. A similar result was obtained by Sausen et al. [83], who reported a ctDNA detection rate of 43% in 51 patients with resectable PDAC.

Based on results published by Tjensvoll et al. [85] ctDNA measurements on KRAS mutations seem to be a marker for monitoring treatment efficacy and PDAC disease progression rather than initial diagnosis. Chen et al. [86] also showed that KRAS mutations in plasma DNA functioned as a strong prognostic factor of survival. Also Marchese et al. [87] found a relatively low sensitivity of KRAS mutations in detecting PDAC. Another current limitation of ctDNA in early PDAC diagnostics seems to be limited consistency in the detection techniques and a lack of technical standardization.

Nevertheless, some investigators have implied that sensitivity and specificity for detection of PDAC can be improved by combining KRAS mutations in blood with an increase in the serum CA19-9 level [79]. For example, Maire et al. [88] reported that the sensitivity and specificity of serum KRAS mutations for the diagnosis of PDAC were 47 and 87%, respectively, whereas the combination of serum KRAS mutations and CA19-9 had a sensitivity and specificity of 98 and 77%, respectively. Moreover, Sefrioui et al. [89] analyzed in his trial a combination of traditional tumor marker CA 19-9 with ctDNA and/or CTCs. The positivity of at least 2 markers was associated with a sensitivity and specificity of 78% and 91%, respectively. As such, CA19-9 in combination with ctDNA and/or CTC analysis may represent an efficient method for diagnosing PDAC.

### 9.3. Exosomes

Exosomes are small vesicles released from the plasma membrane by almost all cells, including cancer cells that have been shown to play an important role in intercellular communication and tumorigenesis [9]. Exosomes carrying various pathogenic miRNAs, mRNAs, DNA fragments, and proteins play an important role in PDAC progression and can be used for the early detection of PDAC [12, 90, 91].

PDAC-derived exosomes enter the circulation at an early stage of cancer development and therefore are promising biomarkers for the early detection of PDAC. However, the method for isolating PDAC related exosomes should be simplified for use in the clinic. Additionally, more evidence from large-scale validation studies is required prior to clinical application.

In summary, the possible role of liquid biopsy (detection of either circulating tumor cells or circulating tumor DNA or tumor exosomes) in early diagnosis of PDAC is in theory very promising. However, data available thus far appear to be conflicting and the real role is unclear. One of the main limitations is a lack of a standardized detection method. Therefore, large-scale validation studies are necessary before clinical application. Overall, findings suggest that higher diagnostic values of liquid biopsy methods available today can be reached when analyzed in a combination with CA19-9.

## 10. Body Fluids

Body fluids and excrements such as saliva, urine, stool, or pancreatic juice can be used to detect PDAC; therefore, it is relevant to consider the role of body fluids in determining biomarkers.

The saliva contains almost the same molecules as the serum because of the high blood flow in salivary glands and it is an easy target for diagnosis of PDAC [12]. Exosomes and miRNAs in saliva could discriminate pancreatic cancer and might be potential biomarkers for detecting PDAC [92, 93].

Urine is an ultrafiltrate of plasma and also may contain valuable biomarkers that could assist with PDAC diagnosis [12]. Patients with early PDAC can be accurately detected by a three-protein biomarker panel (REG1A, TFF1, and LYVE1109) [94] and by the protein NGAL108 [95] in urine. In support of this, a 2015 study reported the use of miRNA in urine also for early detection of PDAC [12, 96].

Similarly, in 2014, Yang et al. [97] showed that miRNAs could be extracted and detected from pancreatic juice and stool efficiently and that miR-21, miR-155, and miR-216 in stool have the potential of becoming biomarkers for screening PDAC.

Disadvantage of investigation of pancreatic juice is the need for upper endoscopy to obtain it. Despite this potential limitation, Wang el al. [3] published a promising data as part of his study focused on profiling miRNAs in pancreatic juice. Results indicated a marked difference in the profiles of four circulating miRNAs (miR-205, miR-210, miR-492, and miR-1427) in pancreatic juice collected from patients with PDAC (miRNAs together predicted PDAC with a specificity of 88% and sensitivity of 87%). Inclusion of serum CA19-9 level further increased the sensitivity to 91% and the specificity to 100%.

In summary, several novel markers (e.g., exosomes, miRNAs, and proteomics) investigated for PDAC detection in blood have also been tested in other types of biological material (e.g., saliva, urine, stool, or pancreatic juice). Initial results seem promising; however, in general they do not appear to exceed the results of particular markers in blood. Furthermore, the use of pancreatic juice for the analyses is hampered by need of upper endoscopy.

## 11. Animal Models 

Early pancreatic cancer is not commonly diagnosed in routine clinical practice and the typical inability to detect the disease before it reaches an advanced stage is one of the reasons for the high mortality rate of PDAC [98]. This is a barrier for studies on early diagnosis of PDAC, for example, by clinical trial. Most of the current reviews are actually differential diagnosis studies and thus diagnostic values of the studied biomarkers for early PDAC are somewhat limited. Animal models represent one of the theoretical solutions. Several types of animal models that can replicate the growth process of PDAC from healthy tissue through PanIN lesions to invasive carcinoma have been developed and used in research [99]. Most of the studies focus on detailed analysis of pathophysiological processes throughout the carcinogenesis and testing the effect of therapeutics. Testing of possible biomarkers of early pancreatic cancer in animal models has been studied in smaller extent but with somewhat positive results. Hingorani et al. have shown specific serum proteomic signature in a mouse model of PanINs that was detectable even in mice with very early stage preinvasive lesions and low overall burden of disease [100].

In summary, animal models of PDAC may be useful modality for research, as early pancreatic cancer is not easy to diagnose in clinical practice and studies in humans are therefore hard to conduct. Some knowledge on PDAC biomarkers has been obtained on animal models, which have suggested a distinctive proteomic profile of premalignant lesions and PDAC. Further research directed at applicability to humans is warranted.

## 12. Conclusion

At present, the only chance of curative treatment for pancreatic cancer is based on prompt diagnosis followed by surgical treatment.

Unfortunately, routine cancer markers (such as CA 19-9) do not seem to be reliable in prediction and detection of early stage of PDAC.

However, there is hope in the area of newly emerging biomarkers of this disease. In particular, the use of combination of these new biomarkers together with traditional CA 19-9 may significantly increase a specificity and sensitivity in early PDAC detection.

While it is hard to predict future development in the field, methods of liquid biopsy, proteomics, metabolomics, and miRNAs appear most promising. The near future probably lies in a carefully selected panel of biomarkers that would allow for earlier diagnosis of PDAC and easier determination of its stage and, ideally, also allow for tailoring of the treatment plan and provide indicator of prognosis/outcome. Therefore, future research inquiries should focus on defining of the precise panel of useful markers and provide clear indications for use in routine daily clinical practice. More research is also vital to identify which of the aforementioned novel markers truly define early PDAC with low risk of metastasizing, as, for example, circulating tumor cells might be the first step in widespread of the disease.

## Figures and Tables

**Table 1:** Overview of biomarkers of early pancreatic cancer.

Traditional biomarkers	CA 19-9, CEA
Proteomics	CEMIP, C4BPA, IGFBP2, IGFBP3

Metabolites	M2-pyruvate kinase (M2-PK), palmitic acid, glucitol, xylitol, inositol, histidine, proline, sphingomyelin, phosphatidylcholine, isocitrate, ceramide

Antibodies	immunoglobulin G4 (IgG4)

Cytokines	interleukin-1*β*, interleukin-6, interleukin-8, interleukin-10, vascular endothelial growth factor and transforming growth factor (macrophage inhibitory cytokine-1 [MIC-1])

Noncoding RNAs (ncRNAs)	microRNAs (miRNAs), small ncRNAs (sncRNAs), long ncRNAs (lncRNAs)

Liquid biopsy	circulating tumor cells (CTCs), circulation tumor DNA (ctDNA) and exosomes

Body fluids	detecting biomarkers from saliva, urine, stool or pancreatic juice

## Abbreviations

AIP: Autoimmune pancreatitis
CA 19-9: Carbohydrate antigen 19-9
CEA: Carcinoembryonic antigen
CEMIP: Cell migration-inducing hyaluronan binding protein
C4BPA: C4b-binding protein *α*-chain
IGFBP2,3: Insulin-like growth factor-binding protein 2,3
ChP: Chronic pancreatitis
M2-PK: M2-pyruvate kinase
MIC-1: Macrophage inhibitory cytokine-1
PDAC: Pancreatic ductal adenocarcinoma
ctDNA: Circulating tumor DNA
cfDNA: Circulating free DNA or also cell-free DNA
CTCs: Circulating tumor cells
IgG4: Immunoglobulin G4
miRNA: Micro-RNA
ncRNA: Noncoding RNA
lncRNA: Long noncoding RNA
KRAS: Kirsten rat sarcoma
PanINs: Pancreatic intraepithelial neoplasms.
